# Supplementary material for: Diffusive excitonic bands from frustrated triangular sublattice in a singlet-ground-state system
Source: Nat Commun. 2023 Apr 12;14:2051. doi: 10.1038/s41467-023-37669-5 (PMC10097669; doi:10.1038/s41467-023-37669-5)
Supplement: Supplementary file 1 — Supplementary Information [file 41467_2023_37669_MOESM1_ESM.pdf]

Supplementary information

I. Photos of a Single Crystal and the Assembly Used in Experiments

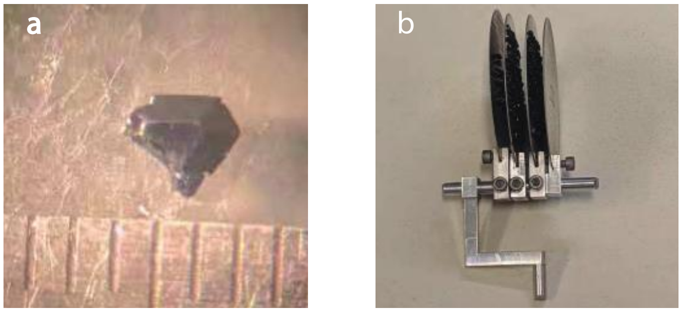

Supplementary Figure 1. Photos of an as-grown single crystal of $Ni_2Mo_3O_8$ and the assembly of single crystals used in neutron scattering experiments. The scale is in mm.

II. X-ray and Neutron Refinements of $Ni_2Mo_3O_8$ and $NiZnMo_3O_8$

We collected over 15,000 reflections on a single crystal of $Ni_2Mo_3O_8$ by X-ray diffraction at Rigaku XtaLAB PRO diffractometer at Spallation Neutron Source (SNS), ORNL. In the refinement, we achieved about 1% (R1 = 1.03%), and there is no evidence of site mixing or deficiency on Ni sites. The space group is $P6_3mc$ and the lattice parameters are $a = b = 5.72781(9)$ Å, $c = 9.83513(16)$ Å as discussed in the main text.

$NiZnMo_3O_8$ will be compared with $Ni_2Mo_3O_8$ to identify the CEF ground states of both materials in discussions to follow. The neutron powder diffraction experiments on $NiZnMo_3O_8$ were performed at room temperature using the high-resolution powder diffractometer BT-1, at NCNR. 5 grams of powder were used. The refined structure has a space group $P6_3mc$ with lattice parameter $a = b = 5.761$ Å, $c = 9.830$ Å. The positions and occupation fractions are refined, yielding 11% disorder for tetrahedral and octahedra sites. The fitting results an R1 = 4.78%. Supplementary Figure 2 shows the refinement results from powder neutron diffraction experiments. Supplementary Table 1 shows the refinement results to be compared with Table 1 for $Ni_2Mo_3O_8$ shown in the main text.

One piece of single crystalline $Ni_2Mo_3O_8$ was aligned in the [*H*, 0, *L*] zone at CORELLI, BL-9, SNS. Magnetic Bragg peaks which can be indexed as (1/2,0,0) are clear in the diffraction pattern (inset of Fig. 1e). The magnetic structure of $Ni_2Mo_3O_8$ was determined from the refinement of 32 magnetic Bragg peaks using the FullProf program. This is consistent with the previous report, except the exact magnetic moments perpendicular and parallel to the *c*-axis are different. The outcome of our refinement is shown in Supplementary Table 2.

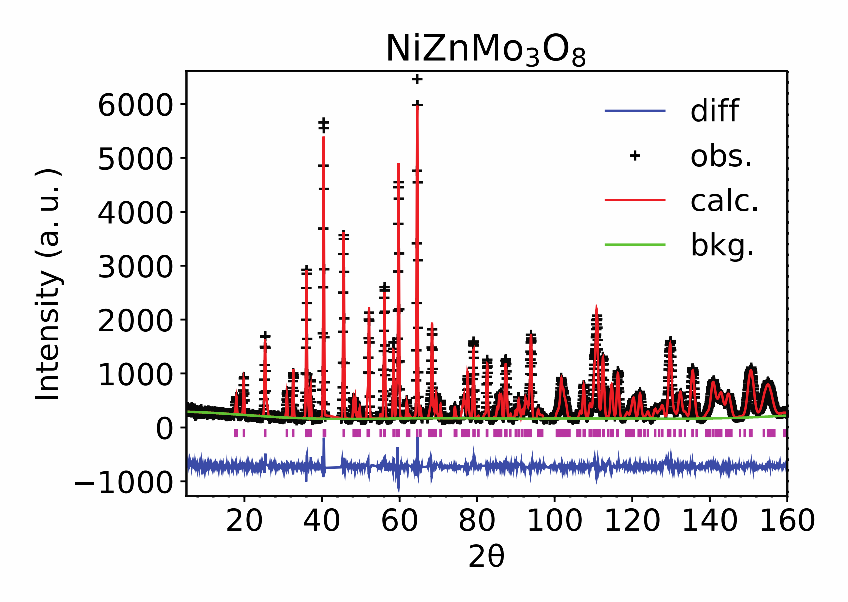

Supplementary Figure 2. Neutron powder diffraction results on $NiZnMo_3O_8$.

Supplementary Table 1. Positions of atoms and occupations in $NiZnMo_3O_8$ as determined from neutron powder diffraction. Ni(1) and Zn(1) represent the tetrahedral site, and Ni(2) and Zn(2) represent the octahedral site.

	x	Y	Z	Occupation
Ni(1)	1/3	2/3	0.9492(1)	0.1192
Ni(2)	1/3	2/3	0.5117(1)	0.8817
Zn(1)	1/3	2/3	0.9492(1)	0.8808
Zn(2)	1/3	2/3	0.5117(1)	0.1183
Mo	0.1462(1)	0.8538(1)	0.2497(1)	1
O(1)	0	0	0.3920(4)	1
O(2)	1/3	2/3	0.1466(5)	1
O(3)	0.4883(3)	0.5117(3)	0.3671(3)	1
O(4)	0.1692(3)	0.8308(3)	0.6334(4)	1

Supplementary Table 2. Refined magnetic moment in the antiferromagnetic state in $Ni_2Mo_3O_8$ at 2 K.

	M_x/μ_B	M_y/μ_B	M_z/μ_B	*ab*-plane/μ_B	angle	Total moment/μ_B
Tetrahedral	1.667	0.833	0.270	1.444	10.605	1.469
Octahedral	1.104	0.552	0.538	0.955	29.404	1.097

III. Susceptibility Measurements with Magnetic Fields along the *c*-axis

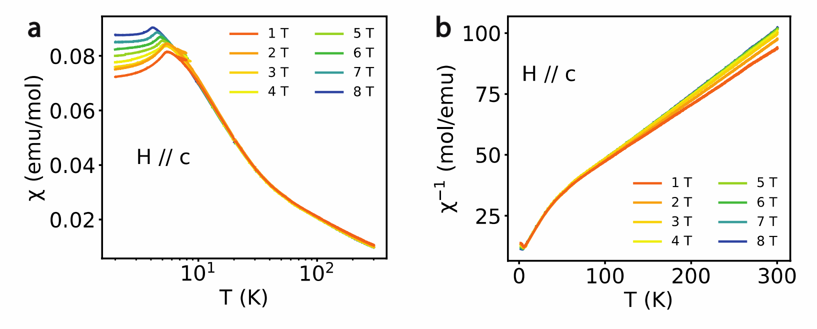

Supplementary Figure 3. The temperature dependence of magnetic susceptibility (a) and inverse susceptibility (b) measured with magnetic fields applied along the *c*-axis.

IV. Crystal Electrical Field (CEF) Analysis

We consider the simplest undistorted cubic crystal field (CF) and the spin orbit coupling (SOC); the level scheme would be:

Octahedral site:

Tetrahedral CF:

$3T_1$ (S=1, L=1 at twice the energy of the $3T_2$ level but with zero dipole intensity)

$3T_2$ (S=1, L=1 orbital triplet excited state at around 200 meV (above 100-500 meV)

$3A_2$ (S=1, L=0 orbital singlet ground state)

SOC does not split the $3A_2$ state.

Tetrahedral site:

Tetrahedral CF:

$3A_2$ (S=1, L=0 orbital singlet excited state at twice energy of the excited $3T_2$ level)

$3T_2$ (S=1, L=1 orbital triplet excited state at 100-500 meV depending on 10 D_q)

$3T_1$ (S=1, L=1 orbital triplet ground state)

SOC splits the $3T_1$ ground state into [2]:

Γ_4 / T (triplet excited state at 75-150 meV depending on SOC but with zero dipole matrix element).

Γ_3 / E (doublet excited state at 50-100 meV depending on SOC but with zero dipole matrix element)

Γ_4 / T (triplet excited state at 15-30 meV depending on SOC integral zeta)

Γ_1 / A (singlet ground state)

Therefore, putting trigonal distortion back, for the octahedral site case, would split the $3A_2$ ground state into a singlet ground state (gs) and doublet excited state (es) [1]. For the tetrahedral site, the gs and 1st es can be a singlet gs and a doublet es [1], or a doublet gs and a singlet es, which depends on the effect of trigonal distortion compared to the SOC.

To identify the scattering around 17 meV, we carried out high-energy CEF levels measurements on $Ni_2Mo_3O_8$, $NiZnMo_3O_8$, and $Zn_2Mo_3O_8$ at 4.5 K using incident neutron energy E_i = 40 meV and 250 meV on SEQUOIA, BL-17, SNS (Supplementary Figure 4). The low-energy CEF

measurements on $Ni_2Mo_3O_8$ and $NiZnMo_3O_8$ were performed at 2.0 K with $E_i = 3.7$ meV at LET, ISIS (Supplementary Figure 5). We used polycrystalline samples of $Ni_2Mo_3O_8$ (6.0 grams), $NiZnMo_3O_8$ (4.24 grams), and $Zn_2Mo_3O_8$ (4.33 grams).

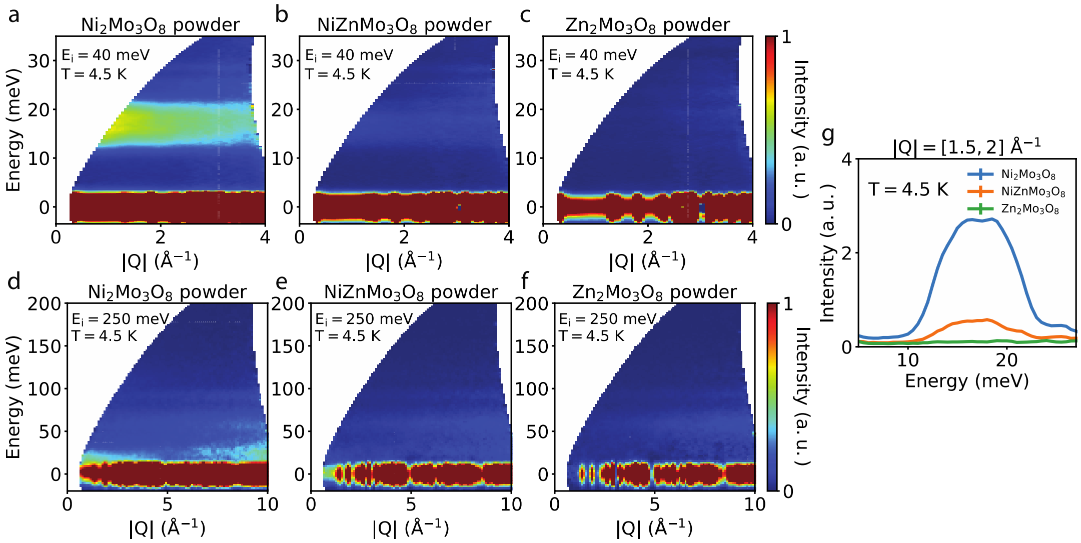

Supplementary Figure 4. Powder spectra and constant-wavevector cut of $Ni_2Mo_3O_8$, $NiZnMo_3O_8$, and $Zn_2Mo_3O_8$ on SEQUOIA, SNS. a-c, Spin excitation spectra of $Ni_2Mo_3O_8$, $NiZnMo_3O_8$, and $Zn_2Mo_3O_8$ at 4.5 K with E_i = 40 meV. d-f, Spin excitation spectra of $Ni_2Mo_3O_8$, $NiZnMo_3O_8$, and $Zn_2Mo_3O_8$ at 4.5 K with E_i = 250 meV. g, Constant-wavevector cuts of panel a-c, which show that the intensity is dramatically reduced when the tetrahedral Ni site is occupied by non-magnetic Zn.

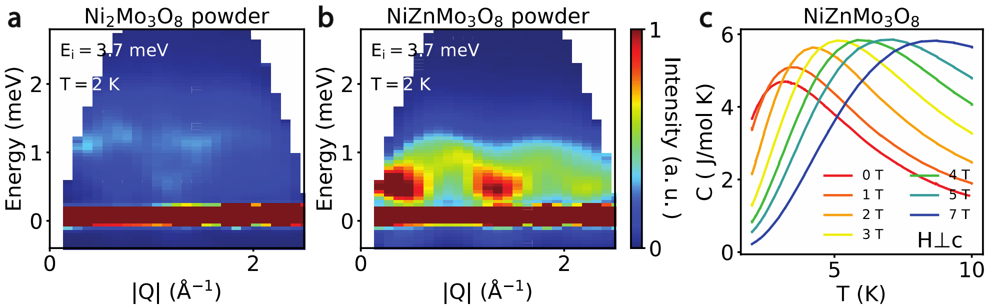

Supplementary Figure 5. Low-energy powder spectra of $Ni_2Mo_3O_8$ and $NiZnMo_3O_8$ on LET, ISIS, and heat capacity data of $NiZnMo_3O_8$. a-b, Spin excitation spectra of $Ni_2Mo_3O_8$ and $NiZnMo_3O_8$ at 2 K with E_i = 3.7 meV. c, Specific heat of $NiZnMo_3O_8$ measured on a single crystal with 0-7 T magnetic fields perpendicular to the c-axis.

Since the intensity of the scattering around 17 meV drops to about 1/10 when about 90% tetrahedral site Ni is replaced by non-magnetic Zn in $NiZnMo_3O_8$ [Supplementary Figure 4g], we conclude that the flat band scattering at 17 meV is totally from the tetrahedral coordinated Ni sites [Supplementary Figure 6a]. Besides, we found at least 3 peaks in the cut (Fig. 2c in the main text)

indicating that the es here is from a doublet. Therefore, both tetrahedral and octahedral Ni sites have a singlet gs and doublet es, consistent with previous predictions [1].

Qualitatively, the singlet-doublet splitting modes for the octahedral site are in low energy region, as the low energy scattering in both $Ni_2Mo_3O_8$ and $NiZnMo_3O_8$ are below 1.5 meV. We fit the heat capacity of $NiZnMo_3O_8$ to a two-level Schottky anomaly model:

$$C_{Schottky} = R(\frac{\Delta}{T})^2 \frac{e^{\Delta/T}}{[1+e^{\Delta/T}]^2}, \quad (1)$$

and get Δ = 0.8 meV. The schematics of CEF for both sites are shown in Supplementary Figure 6.

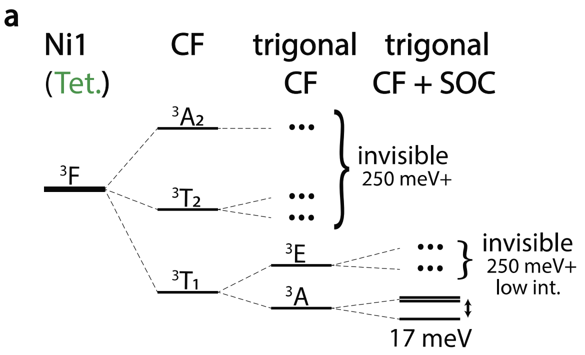

Supplementary Figure 6. Schematics of crystal field levels of tetrahedral and octahedral Ni sites.

Based on the point-group symmetry at the Ni^{2+} atomic site and using the Stevens operator formalism, the Hamiltonian of the CEF for both sites is $H_{CEF} = B_2^0\hat{O}_2^0 + B_4^0\hat{O}_4^0 + B_4^3\hat{O}_4^3$, where B_n^m (m and n are integers and $n \geq m$) are CEF parameters that will be determined experimentally, and the Stevens operators $\hat{O}_n^m$ are polynomial functions of the components of the total angular momentum operator J_z, J_+, and J_- ($J_\pm = J_x \pm iJ_y$).

For the octahedral site, we fit the CEF parameters to the 0.8 meV spin gap and magnetic susceptibility data of $NiZnMo_3O_8$, resulting $B_2^0 = 1.0\,meV$, $B_4^0 = -0.5\,meV$, and $B_4^3 = 30\,meV$, which gives the first excited doublet at 0.78 meV and the second excited doublet at 634 meV. The fitting for the tetrahedral site is more sophisticated due to the intervention of two magnetic Ni sites in the susceptibility data of $Ni_2Mo_3O_8$. A rough fit that mainly depends on the 17 meV spin gap yields $B_2^0 = 7.0\,meV$, $B_4^0 = -0.7\,meV$, and $B_4^3 = 2.2\,meV$, with the first excited doublet at 17.0 meV and the second excited doublet at 201 meV.

V. L-dependence and Temperature dependence of spin excitations in the high energy range

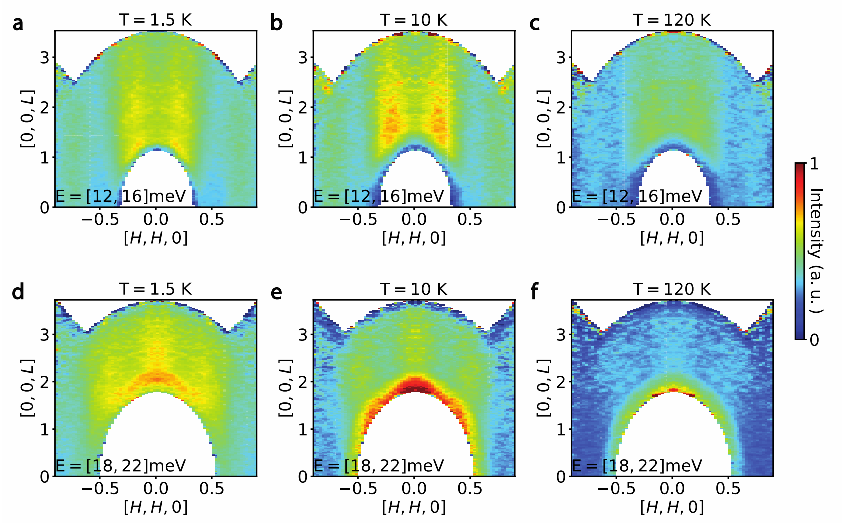

Supplementary Figure 7. Momentum and temperature dependence of magnetic scattering in $Ni_2Mo_3O_8$ measured with the assembly of single crystals on SEQUOIA. a-c, Momentum dependence of the magnetic scattering at 1.5 K, 10 K, and 120 K corresponding to Fig. 3i, 3j, and 3k, respectively. The energy integration range is 12 meV - 16 meV. i-k, Momentum dependence of the magnetic scattering at 1.5 K, 10 K, and 120 K corresponding to Fig. 3f, 3g, and 3h, respectively. The energy integration range is 18 meV - 22 meV. Data in both energy ranges show no L-modulation, indicating a 2D effective mode for high-energy spin excitations.

Supplementary Figure 7 shows the *L*-dependence of spin excitations for the tetrahedral sites, revealing that spin excitations are weakly *L*-dependent and better defined above the TN at 10 K. This is consistent with the notion that spin excitations in these energy ranges are 2D.

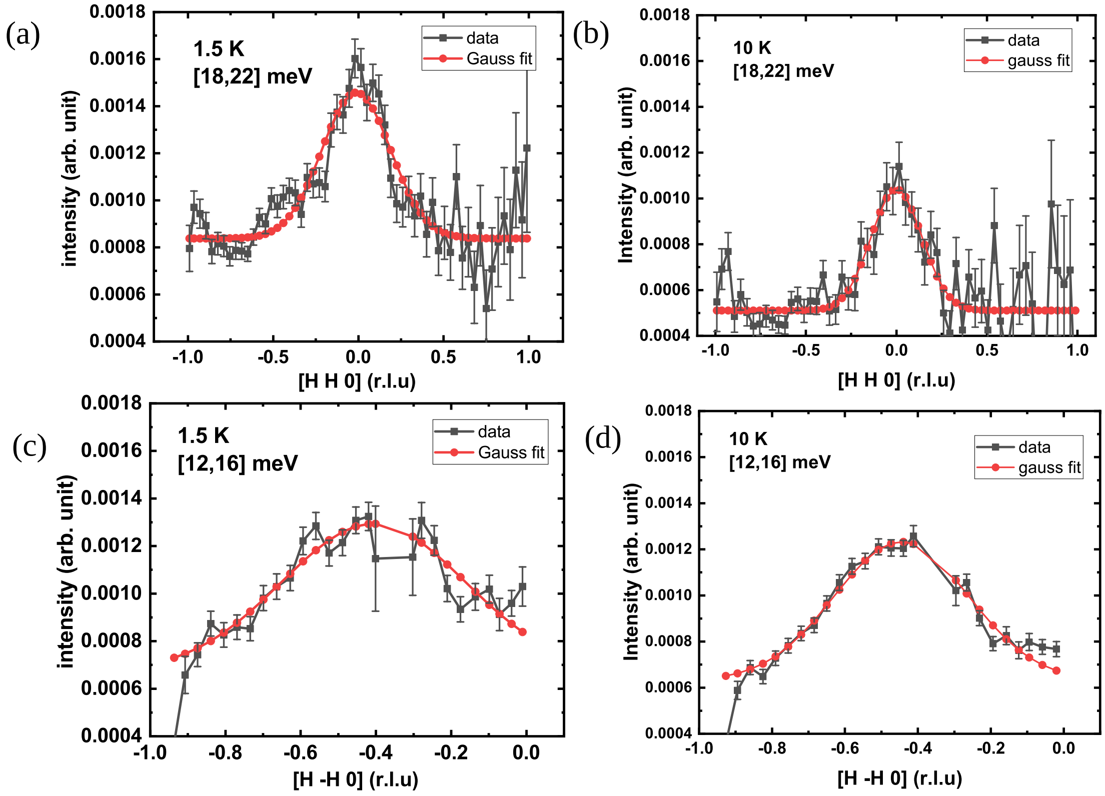

Supplementary Figure 8. Spin-spin correlations of the CEF excitations below and above T_N. a-d panels show the Q cut of magnetic scattering in Figs. 3f, 3g, 3i & 3j, and their Gaussian fits, respectively. For a & b, i.e. in the high energy range [18,22] meV, the cuts are along [H H 0] direction and the integration range of [H -H 0] is from -0.01 to 0.01 r.l.u. For c & d, i.e. in the low energy range [12,16] meV, the cuts are along [H -H 0] direction and the integration range of [H H 0] is from -0.01 to 0.01 r.l.u. The FWHM from the Gaussian fits is 0.58 and 0.33 r.l.u for a & b, 0.52 and 0.40 r.l.u for c & d, which show sharper peaks above T_N. The spin correlation lengths calculated from the Gaussian fit are 4.38, 7.70, 8.46, and 11.01 Å for panels a-d.

VI. Low energy spin waves in $Ni_2Mo_3O_8$

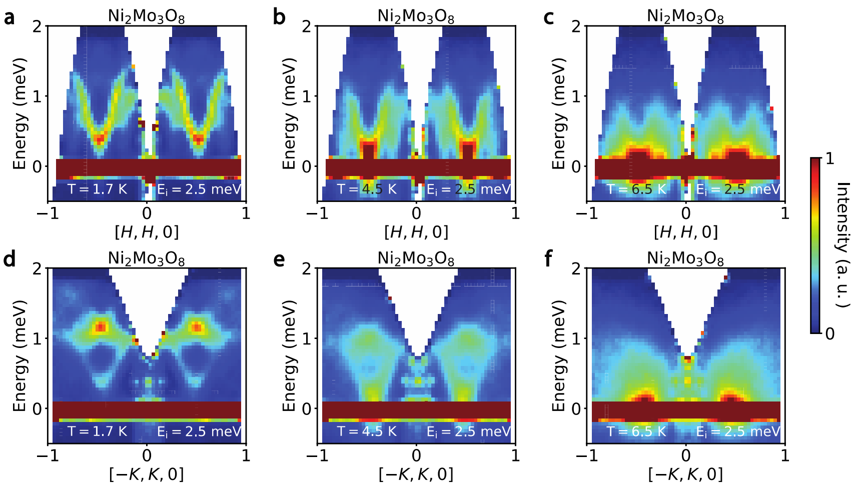

Supplementary Figure 9. Low-energy spin wave excitations in $Ni_2Mo_3O_8$ measured with single crystals on CNCS, SNS. a-c, Spin excitations with E_i = 2.5 meV at 1.7 K, 4.5 K and 6.5 K plotted along the [*H*, *H*, *0*] direction. d-f, Spin excitations with E_i = 2.5 meV at 1.7 K, 4.5 K, and 6.5 K plotted along the [-*K*, *K*, *0*] direction. The low energy spectra cannot be fit by linear spin wave theory with a 3rd nearest neighbor exchange Hamiltonian with anisotropy and DM terms.

VII. Cluster Form Factor Calculation

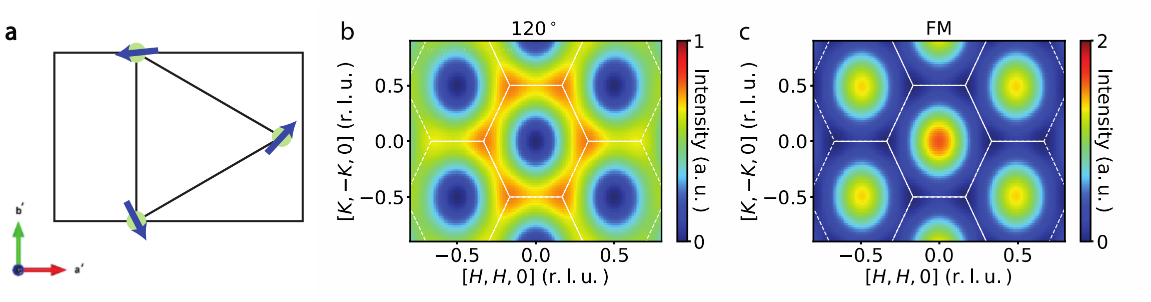

Supplementary Figure 10. Schematic of a spin cluster and $F_{eff} * F_{Ni}^2$ for 120° and FM configurations.

We will discuss 120° and ferromagnetic (FM) configurations in this section. We first consider a 120° spin configuration on 3 Ni atoms sitting on the orthorhexagonal lattice with the following coordinates: $\boldsymbol{S_0}$ at $(\frac{1}{3}, 0)$, $\boldsymbol{S_1} = \boldsymbol{S_0}\, R_{1/3}$ at $(-\frac{1}{6}, \frac{1}{2})$, and $\boldsymbol{R_1} = \boldsymbol{S_0}\, R_{1/3}^2$ at $(-\frac{1}{6}, -\frac{1}{2})$. The rotation matrix is defined as:

$$R_{1/3} = \begin{bmatrix} -\frac{1}{2} & -\frac{\sqrt{3}}{2} \\ \frac{\sqrt{3}}{2} & -\frac{1}{2} \end{bmatrix}. \quad (3)$$

The Fourier transform of the spin on this cluster is:

$$\boldsymbol{S_c}(h,k,l) = \sum_{j=0}^{2} \boldsymbol{S_0}\, R_{1/3}^{j} F_{Ni}(|Q_{hkl}|) exp\left[-i2\pi\left(x_j h + y_j k\right)\right] = \boldsymbol{S_0}\mathbb{M}F_{Ni}(|Q_{hkl}|). \quad (4)$$

Here the matrix $\mathbb{M}$ has the following form:

$$\mathbb{M} = \begin{bmatrix} \boldsymbol{M_{11}} & \boldsymbol{M_{12}} \\ -\boldsymbol{M_{12}} & \boldsymbol{M_{11}} \end{bmatrix}, \tag{5}$$

$$\boldsymbol{M_{11}} = \exp[-i2\pi\frac{h}{3}] - \frac{1}{2}\boldsymbol{exp}[-i2\pi(-\frac{h}{6}+\frac{k}{2})] - \frac{1}{2}\boldsymbol{exp}[-i2\pi(-\frac{h}{6}-\frac{k}{2})], \tag{6}$$

$$\boldsymbol{M_{12}} = -\frac{\sqrt{3}}{2}\exp[-i2\pi\left(-\frac{h}{6}+\frac{k}{2}\right)] + \frac{\sqrt{3}}{2}\exp[-i2\pi\left(-\frac{h}{6}-\frac{k}{2}\right)]. \tag{7}$$

The magnetic contribution to the observable $S(Q,E)$ is proportional to the term $|\hat{\kappa} \times (\boldsymbol{S_c} \times \hat{\kappa})|^2$, where $\hat{\kappa} \equiv \boldsymbol{Q}/Q$. Now if the only constraint on the spin configuration of the cluster is the 120° rotation, and the direction of $\boldsymbol{S_0}$ is free to choose, on average we have

$$S(Q,E) \propto |\boldsymbol{S_c}|^2 = \boldsymbol{S_0}\ \mathbb{M}\mathbb{M}^\dagger \boldsymbol{S_0}^\dagger F_{Ni}^2. \tag{8}$$

The off-diagonal terms of the matric $\mathbb{M}\mathbb{M}^\dagger$ are only differ by a negative sign, which will cancel out the $S_{0x}S_{0y}$ terms on the right-hand side. Meanwhile, the diagonal term of the metric has the same expression of $|\boldsymbol{M_{11}}|^2 + |\boldsymbol{M_{12}}|^2$, therefore the scattering function becomes:

$$S(Q,E) \propto |\boldsymbol{S_c}|^2 = \boldsymbol{S_0^2}(|\boldsymbol{M_{11}}|^2 + |\boldsymbol{M_{12}}|^2)F_{Ni}^2. \tag{9}$$

We define $|\boldsymbol{M_{11}}|^2 + |\boldsymbol{M_{12}}|^2$ as F_{eff}, the effective form factor. Substituting the expressions of $\boldsymbol{M_{11}}$ and $\boldsymbol{M_{12}}$, we have

$$F_{eff} = 3 - \cos\pi(h-k) - \cos\pi(h+k) - \cos 2\pi k. \tag{10}$$

After taking lattice symmetry into account, the result is plotted in Supplementary Figure 10b. Now we consider the FM spin configuration. When the spins are colinear, the 2D identity matrix $\mathbb{M} = M \cdot \mathbb{1}$. Similarly, we have

$$F_{eff} = |\boldsymbol{M_{11}}|^2 = 3 + 2\cos\pi(h-k) + 2\cos\pi(h+k) + 2\cos 2\pi k. \tag{11}$$

Supplementary Figure 10c shows the results of the FM configuration. As we can see from the effective form factor, the two patterns are complementary to each other.

VIII. Flavor-Wave Theory

For the spin-1 system, there is a SU(3) symmetry from the rotation among three spin components with the quantum numbers $S^z = -1,0,+1$. One can formulate the low-energy excitations using the SU(3) flavor-wave theory. Let us consider the following three linearly independent states for the spin-1 Hilbert space

$$|x\rangle = -i|S^x = 0\rangle = \frac{-i}{\sqrt{2}}(|S^z = -1\rangle - |S^z = +1\rangle), \tag{12}$$

$$|y\rangle = |S^y = 0\rangle = \frac{1}{\sqrt{2}}(|S^z = -1\rangle + |S^z = +1\rangle), \tag{13}$$

$$|z\rangle = |S^z = 0\rangle. \tag{14}$$

We introduce three bosonic partons b_x, b_y, b_z (on each site) such that $|\mu\rangle = b_\mu^\dagger|\emptyset\rangle$ where $\mu = x$, y, z, and $|\emptyset\rangle$ is the boson vacuum. The spin operator can be nicely represented by the partons

$$S^\mu = -i\,\varepsilon^{\mu\nu\rho} b_\nu^\dagger b_\rho. \tag{15}$$

To get back to the Hilbert space, there is a local gauge constraint $b_x^\dagger b_x + b_y^\dagger b_y + b_z^\dagger b_z = 1$. We would like to describe the mean-field state that is experimentally observed. The strategy will be to carry out a unitary rotation of the boson operators to define another set of bosons a_x, a_y, a_z

$$\begin{pmatrix} a_x^\dagger \\ a_y^\dagger \\ a_z^\dagger \end{pmatrix} = \begin{pmatrix} sin(\beta)\, cos(\theta) - i\, sin(\alpha)\, cos(\beta)\, sin(\theta) & -cos(\beta)\, cos(\theta) - i\, sin(\alpha)\, sin(\beta)\, sin(\theta) & i\, cos(\alpha)\, sin(\theta) \\ cos(\alpha)\, cos(\beta) & cos(\alpha)\, sin(\beta) & sin(\alpha) \\ -sin(\alpha)\, cos(\beta)\, cos(\theta) + i\, sin(\beta)\, cos(\theta) & -sin(\alpha)\, sin(\beta)\, cos(\theta) - cos(\beta)\, sin(\theta) & cos(\alpha)\, cso(\theta) \end{pmatrix} \begin{pmatrix} b_x^\dagger \\ b_y^\dagger \\ b_z^\dagger \end{pmatrix} \tag{16}$$

Accordingly, the local gauge constraint becomes $a_x^\dagger a_x + a_y^\dagger a_y + a_z^\dagger a_z = 1$. By a suitable choice of $(\alpha_j,\ \beta_j,\ \theta_j)$ on each site $\boldsymbol{j}$, we can arrange that the mean-field ground state is

$$|\Psi_{\mathrm{MF}}\rangle = \prod_{\mathrm{j}} \mathrm{a}_{\mathrm{j,z}}^\dagger |\emptyset\rangle, \tag{17}$$

which gives the expectation values of the spin operator and the anisotropy

$$\langle S_j \rangle = sin\,(2\theta_j)(\,cos\,(\alpha_j)\,cos\,(\beta_j), cos\,(\alpha_j)\,sin\,(\beta_j), sin\,(\alpha_j)), \tag{18}$$

$$\left\langle \left(S_j^z\right)^2 \right\rangle = \sin^2(\theta_{\mathrm{j}})(1 + \cot^2(\theta_j)\,\sin^2(\alpha_j)). \tag{19}$$

What is nice about the approach is that we can use it to obtain the flavor wave expansion around the mean field state. We condense the boson a_z and strictly impose the gauge constraint by

$$\langle a_z \rangle = \langle a_z^\dagger \rangle = \left(1 - a_x^\dagger a_x - a_y^\dagger a_y\right)^{1/2} \tag{20}$$

Inserting this back into the representation for the spin operators, we obtain a still exact but non-analytic representation of the problem in terms of two bosons per site, which is analogous to the Holstein-Primakoff representation (but for SU(3)). By analogy, the flavor wave expansion is obtained by expanding the resulting expressions in powers of the two remaining boson fields, which amounts to expanding the square root term.

We take the sublattice structure of the four-sublattice antiferromagnet. Specifically, the four sublattices can be delineated by subscripts $t1$, $t2$, $o1$, $o2$, where $t1$, $t2$ are the two tetragonal sites and $o1$, $o2$ are the two octahedral sites in the four-site unit cell, and $t2$, $o2$ are time-reversal conjugates of $t1$, $o1$. We introduce θ_j, α_j , β_j such that

$$\langle \boldsymbol{S}_{t1} \rangle = -\langle \boldsymbol{S}_{t2} \rangle = \sin(2\theta_t)\ (0, \cos(\alpha_t), sin(\beta_{\mathrm{t}})), \tag{21}$$

$$\langle \boldsymbol{S}_{o2} \rangle = -\langle \boldsymbol{S}_{o1} \rangle = sin(2\theta_o)\ (0, cos(\alpha_o), sin(\beta_o)), \tag{22}$$

where $\beta_t = \beta_o = \pi/2$. With the Eq. 15 and Eq. 16, we can express the Hamiltonian in terms of a_x, a_y, a_z. Now insert Eq. 20 to obtain a non-linear form in terms of a_x, a_y bosons. We then series expand up to quadratic order in these operators. The zeroth order term represents the mean-field energy parametrized by the angles

$$\frac{E_0}{N} = -2\, J_1 \sin(2\theta_t)\, \sin(2\theta_o)\, ((1+\gamma/2)\cos(\alpha_t)\cos(\alpha_o) + sin(\alpha_t)\sin(\alpha_o) + 2d\, sin(\alpha_o - \alpha_t)) \tag{23}$$

$$-J_t\big(1 - cos(4\theta_t)\big) - J_o\big(1 - cos(4\theta_o)\big) - 6\, J_3\, cos(\alpha_t - \alpha_o)\, sin(2\theta_t) sin(2\theta_o) \tag{24}$$

$$+2D_t \sin^2(\theta_t)\,(1 + \cot^2(\theta_t)\sin^2(\alpha_t)) + 2D_o \sin^2(\theta_o)\,(1 + \cot^2(\theta_o)\sin^2(\alpha_o)), \tag{25}$$

where N is the total number of four-sublattice unit cells. Given a set of parameters J_1, γ, D_t, D_o, J_t, J_o, J_3, d, the angles θ_t, θ_o, α_t, α_o can be obtained by variationally minimizing E_0.

Under the condition that the mean field state is a local energy minimum, the linear term in the boson operators vanishes, and one obtains a quadratic form. After Fourier transforming to momentum space in the magnetic Brillouin zone, it is conveniently expressed by defining the field $\Psi_k = (\vec{a}_{\boldsymbol{k}}, \vec{a}^\dagger_{-\boldsymbol{k}})$ where

$$\vec{a}_{\boldsymbol{k}} = (a_{x,t,1,k}, a_{y,t,1,k}, a_{x,t,2,k}, a_{y,t,2,k}, a_{x,o,1,k}, a_{y,o,1,k}, a_{x,o,2,k}, a_{y,o,2,k}). \tag{26}$$

Then we have the bosonic Bogoliubov-de Gennes (BdG) Hamiltonian

$$\mathrm{H} = \mathrm{E}_0 + \sum_k \frac{1}{2} \Psi_k^\dagger \mathcal{H}_k^{BdG} \Psi_k, \qquad \mathcal{H}_{\boldsymbol{k}}^{BdG} = \begin{pmatrix} A_{\boldsymbol{k}} & B_{\boldsymbol{k}} \\ B^*_{-\boldsymbol{k}} & A^*_{-\boldsymbol{k}} \end{pmatrix}, \tag{27}$$

where $A_{\boldsymbol{k}}$, $B_{\boldsymbol{k}}$ are 8×8 matrices satisfying $A_{\boldsymbol{k}} = A^T_{\boldsymbol{k}}$ and $B_{\boldsymbol{k}} = B^T_{-\boldsymbol{k}}$. The flavor-wave energy spectrum is calculated by numerically diagonalizing $g\mathcal{H}_{\boldsymbol{k}}^{BdG}$, where $g = diag(1,\ldots,1,-1,\ldots,-1)$. Finally, the structural factor can be evaluated using the BdG Green function of the flavor-wave excitations

$$G(iw, k) = \left[i\omega g - \mathcal{H}_{\boldsymbol{k}}^{BdG}\right]^{-1}. \tag{28}$$

The retarded/advanced Green function is given by the analytical continuation $i\omega \to \omega \pm i\,\Gamma$, where we set the phenomenological damping Γ as the half-bandwidth of high energy excitations. In Fig. 4 of the main text, we have used the parameters

$$D_t = 16\, meV, D_o = 1.0\, meV, d = 0.3, \gamma = 0.5,$$
$$J_1 = 2.0\, meV, J_t = 0.5\, meV, J_o = 0.15\, meV, J_3 = 0.0\, meV.$$

Reference:

[1] Morey, Jennifer R., et al. "$Ni_2Mo_3O_8$: Complex antiferromagnetic order on a honeycomb lattice." *Physical review materials* 3.1 (2019): 014410.
[2] Weakliem, Herbert A. "Optical spectra of Ni^{2+}, Co^{2+}, and Cu^{2+} in tetrahedral sites in crystals." *The Journal of Chemical Physics* 36.8 (1962): 2117-2140.
